# Erector Spinae Plane block with fascial plane catheter for the Early Analgesia of Rib fractures in trauma (ESPEAR): a multicentre feasibility randomised trial

**DOI:** 10.1016/j.bjao.2025.100498

**Published:** 2025-10-13

**Authors:** David W. Hewson, Jessica Nightingale, Reuben Ogollah, Adam Brooks, Lauren Blackburn, Benjamin J. Ollivere, Matthew L. Costa, Tim Egan, Peter Bates, Ian Tyrrell-Marsh, Nigel M. Bedforth

**Affiliations:** 1 Anaesthesia and Critical Care Medicine, Queen’s Medical Centre, Nottingham University Hospitals NHS Trust, Nottingham, UK; 2 Anaesthesia and Critical Care, Academic Unit of Injury, Recovery and Inflammation Sciences, School of Medicine, University of Nottingham, Nottingham, UK; 3 Orthopaedic and Trauma Surgery, Queen’s Medical Centre, Nottingham University Hospitals NHS Trust, Nottingham, UK; 4 Nottingham Clinical Trials Unit, Applied Health Research Building, University Park, University of Nottingham, UK; 5 East Midlands Major Trauma Centre, Queen’s Medical Centre, Nottingham University Hospitals NHS Trust, Nottingham, UK; 6 Orthopaedic and Trauma Surgery, Academic Unit of Injury, Recovery and Inflammation Sciences, School of Medicine, University of Nottingham, Nottingham, UK; 7 Nottingham NIHR Musculoskeletal Biomedical Research Centre, University of Nottingham, Nottingham, UK; 8 Oxford Trauma and Emergency Care, Nuffield Department of Orthopaedics, Rheumatology and Musculoskeletal Sciences, University of Oxford, Oxford, UK; 9 Anaesthesia and Critical Care Medicine, Royal London Hospital, Bart’s Health, London, UK; 10 Orthopaedic Surgery, Royal London Hospital, Bart’s Health, London, UK; 11 Department of Anaesthesia and Critical Care Medicine, Manchester Royal Infirmary, Oxford Road, Manchester, UK

**Keywords:** analgesia, chest trauma, erector spinae plane block, regional anaesthesia, rib fractures

## Abstract

**Background:**

Traumatic rib fractures cause significant acute pain. Patients are at risk of hypoventilation, atelectasis, hypoxia, retained secretions, pneumonia, respiratory failure, and death. Effective analgesia is thought to reduce these adverse outcomes. There is widespread variation in analgesic treatments given to patients including oral, intravenous, and epidural routes of administration. Erector spinae plane (ESP) block, a novel regional analgesic technique, may be effective, but high-quality evidence is lacking.

**Methods:**

To determine if a definitive trial of ESP block and catheter in rib fractures is possible, we conducted a multicentre, randomised, controlled pilot study with feasibility assessment. Adults with rib fractures were randomised in a 1:1 ratio to either (i) ESP blockade and catheter, or (ii) placebo ESP blockade and catheter, both for 72 h. In addition, all participants received multimodal analgesia. Participants and outcome assessors were blinded. The primary feasibility outcomes were recruitment rate (target: ≥1.11 participants/site/month), retention rate (target: ≥80%), and trial acceptability assessed by staff interview. Pre-specified red–amber–green criteria were agreed to determine feasibility of a future definitive clinical trial on this topic.

**Results:**

Twenty-five participants (mean [standard deviation] age 57 [16] yr, number of rib fractures 5 [3]) were recruited from three UK major trauma centres at a rate of 0.69 participants per site per month. Retention to 6-week follow-up was 80%. Based on our criteria, the current study design is not feasible for adoption into a definitive trial. For future research in this area, we recommend substantial modification to (i) the intervention, (ii) means of bias reduction, and (iii) timing and nature of outcome measure assessments.

**Conclusions:**

Based on pre-specified criteria, a definitive examination of the clinical effectiveness of ESP block in the analgesic management of adults after blunt force chest wall injury is only feasible if substantial amendments to trial processes piloted in this study are undertaken. An open-label assessment of single-shot ESP block, applying patient-reported average pain intensity of the first 24 h as the primary outcome, and conducted at sites with established ESP analgesic pathways, may overcome the most significant feasibility barriers identified by the present study.

**Clinical trial registration:**

ISRCTN49307616.

Rib fractures after blunt chest wall trauma can be associated with severe pain, impairing effective cough and preventing deep breathing.^1^ Resultant atelectasis and retained secretions can cause hypoxaemia, pneumonia, and/or progressive respiratory failure.^2^^,^^3^ Underlying lung contusions may lead to pulmonary oedema and/or haemorrhage, and result in poor compliance and gas exchange. A key clinical objective is the provision of effective analgesia for patient comfort and to facilitate normal respiration and cough, thereby minimising the risk of respiratory failure.^4^ Despite this, there is no agreement about optimal analgesic regimes beyond a preference for multimodal approaches.^5^ Thoracic epidural analgesia (TEA) provides excellent pain relief, but is associated with significant adverse events, requires considerable technical skill for insertion, and necessitates admission to high-acuity hospital facilities for safe monitoring.^6^^,^^7^ As a result, TEA is being supplanted in many centres by fascial plane blocks including the erector spinae plane (ESP) block.^8^ The anatomical basis, and controversies, of ESP block have been expertly reviewed elsewhere.^9^ The role of ESP block in the management of acute rib fracture pain remains uncertain. Although observational and single-centre experimental studies signal efficacy, there is a critical paucity of experimental pragmatic multicentre trials published in this setting.

High-quality definitive clinical evidence is urgently needed to assist clinicians on whether the ESP block is an effective component of multimodal analgesia regimes in patients with rib fractures. A definitive trial on this topic would promote evidence-based practice in rib fracture management and reduce unnecessary variation in clinical practice between centres. Undertaking a multicentre trial of ESP block in this setting raises important practical concerns including: timely access to potential participants taking into account emergency clinical pathways, availability of trained anaesthetists to deliver ESP blocks with fidelity and reproducibility, willingness of clinicians and potential participants to undergo randomisation, effectiveness of any proposed system of blinding to treatment allocation, the burden on participants and researchers of candidate outcome measures, and the ability of researchers to undertake timely outcome assessments in a high-acuity inpatient environment.

The aim of this study is to determine if it is feasible to undertake a definitive multicentre randomised controlled trial to establish if ESP block with fascial plane catheter is a clinically effective early treatment for acute pain in patients hospitalised with rib fractures. Formal hypothesis testing for effectiveness or efficacy is not undertaken in feasibility studies. The objective of this study is not to assess efficacy or effectiveness but to test and refine a definitive research protocol by (i) establishing recruitment and retention rates, (ii) providing data on candidate clinical outcome measures including completeness of reporting, and (iii) determining barriers and facilitators to study engagement and acceptability of study interventions and assessments to participants, clinicians, and researchers.

## Methods

Erector Spinae Plane block with fascial plane catheter for the Early Analgesia of Rib fractures in trauma (ESPEAR) is an investigator-initiated, multicentre, parallel group, feasibility trial, reported according to CONSORT guideline extension for feasibility trials.^10^ The trial protocol and materials were approved by the Oxford B NHS Research Ethics Committee (reference: 22/SC/0005; date: 22 February 2022). The full trial protocol was published open access^11^ and a summary registered on a publicly accessible database (ISRCTN Registry: 49307616; date: 9 March 2022). The study was conducted at three large UK major trauma centres: Manchester Royal Infirmary, Queen’s Medical Centre, Nottingham, and the Royal London Hospital. These sites were selected after site screening as having, respectively, higher, medium, and lower use of ESP block for analgesia after rib fractures in their pre-existing standard care clinical pathways, reflecting the current variability in NHS care and designed to assist in site selection decisions in any future definitive trial.

### Eligibility criteria

Inclusion criteria were: age ≥18 yr, mechanism of injury after blunt thoracic trauma, radiographic evidence of one or more new traumatic rib fractures, moderate or severe unilateral acute pain (defined as 11-point numerical rating scale pain >4 with the patient performing a vital capacity breath or attempting effective cough) at time of enrolment. Exclusion criteria were: patient refusal or inability to give informed written consent for any reason, thoracic injury requiring emergent operative or interventional radiology management, allergy to local anaesthetic, infection at site of ESP block, or actual or estimated total body weight ≤50 kg.

### Enrolment

Clinical staff at participating major trauma centres identified potential participants who met study eligibility criteria. After assessment of eligibility, informed written consent was obtained by a member of the research team.

Randomisation in a 1:1 allocation ratio was performed by site research staff using a secure web-based automated computer-generated minimisation algorithm with treatment groups balanced for: age, gender, polytrauma, and unilateral or bilateral rib fractures. Randomisation was to two groups:

1. ESP block. Performed by an anaesthetist with the participant in a seated position under aseptic technique according to the method described by Kot and colleagues.^12^ An ultrasound-guided 20–40 ml injection of long-acting local anaesthetic was performed at the vertebral level of maximum rib pain. An ESP catheter was then inserted under the ESP muscle, affixed to the patient using skin glue, dressings, or both. The catheter was connected to an infusion of long-acting local anaesthetic on either a programmable intermittent bolus or, if a programmable intermittent bolus regime was not available at the site, a fixed-rate infusion system for 72 h.
2. Ultrasound-guided placebo ESP block. Performed by an anaesthetist with the participant in a seated position under aseptic technique. An ultrasound-guided 3 ml sub-dermal injection of saline 0.9% was performed at the vertebral level of maximum rib pain. A placebo ESP catheter was then affixed externally to the patient using skin glue, dressings, or both. The catheter was connected to an infusion of low-dose local anaesthetic on either a programmable intermittent bolus or fixed-rate infusion system for 72 h, however the infusion device was left in an ‘off’ mode for blinding purposes.

Participants in both groups received supportive therapy and multimodal analgesia consistent with local site implementation of 2016 British Orthopaedic Association guidelines at the discretion of their usual care team.^13^ Participants could go on to receive additional regional anaesthetic techniques (for example TEA insertion) if deemed clinically indicated by their usual care team. Participants and researchers undertaking outcome assessments were blinded to study group allocation. Anaesthetists siting blocks and all other clinical staff caring for participants were unblinded but otherwise uninvolved in study processes.

### Primary feasibility outcomes

Applying data from a comparable trial assessing acute surgical intervention in adult patients with rib fractures, we set our target recruitment rate to be ≥1.11 participants randomised per site per month. We established *a priori* criteria for definitive trial feasibility based on the core issues of recruitment rate, retention rate, and the qualitative assessment of study barriers and facilitators reported by participants, clinicians, and researchers.^14^ Our red–amber–green feasibility thresholds are defined in Table 1.

**Table 1:**
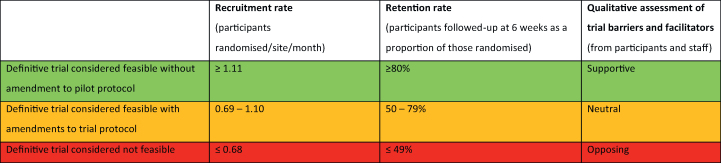
Pre-specified red–amber–green (RAG) feasibility criteria.

### Secondary feasibility outcomes

Secondary feasibility outcomes, not incorporated into the thresholds of Table 1, but informative for any future definitive trial were the trial eligibility rate (proportion of patients screened who are eligible for enrolment) and consent rate (proportion of eligible patients who provide consent for inclusion and were randomised). In addition, the availability and willingness of anaesthetists to randomise participants and site trial interventions was assessed by semi-structured interviews conducted by the central research team.

### Secondary clinical outcomes

Pre-injury medical and drug history and injury characteristics were recorded at baseline before trial intervention. Chest wall pain intensity (at rest and functional pain during deep breathing or coughing), opioid consumption, supplemental oxygen requirement, bedside spirometry (forced expiratory volume in 1 s, vital capacity), complications of ESP block (dislodgement, blocked catheter, technical failure, bleeding or infection at catheter site, local anaesthetic systematic toxicity) and requirement for additional regional anaesthetic techniques to assist in pain management were recorded at baseline and at 3, 6, 9, 12, 24, 48, and 72 h after trial intervention. Length of stay in hospital facilities (levels 0, 1, 2, 3), new post-injury diagnosis of pneumonia, health-related quality of life, and all-cause mortality were recorded at 6 weeks. Outcome assessment time points 3, 6, 9, and 12 h after trial intervention were described as ‘hyperacute’ and were anticipated to significantly fall due in the 19:00–07:00 period, assuming that the trial intervention was delivered within the 07:00–19:00 period. Outcome assessment time points at 12, 48, and 72 h were described as ‘acute’.

### Sample size

Formal sample size calculation for between-group comparisons is not appropriate for feasibility studies. To answer our key objectives, we aimed to recruit 50 participants to allow estimation of recruitment and retention rates with a margin of error of <10%.

### Statistical analysis

Data were collected via a REDCap database (Research Electronic Data Capture, Vanderbilt University, Nashville, TN, USA). Data analysis was descriptive {median (inter-quartile range [IQR] for ordinal; mean (standard deviation) for continuous; raw count (number, %) for nominal data} relating to feasibility objectives and candidate clinical outcomes and narrative for qualitative assessments of study barriers, facilitators, and acceptability. No missing data were imputed.

### Qualitative methodology

The purpose of the qualitative assessment was to enrich the quantitative data collected by understanding the acceptability and feasibility of delivering the trial interventions in clinical practice and exploring patient acceptability of interventions and trial experiences. A flexible topic guide supported semi-structured interviews of participants, potential participants who declined consent for trial inclusion, site clinicians, and site researchers. These interviews were undertaken by a member of the research team (JN) and explored barriers to recruitment and the acceptability, value, benefit, and harms of the intervention and comparator.

Separate additional informed written consent was obtained from patients for these interviews. Staff participants were asked to provide verbal consent at the point of interview. Interviews were conducted either face-to-face, via telephone or video-link (Microsoft Teams) depending on individual preference. After interview, data were transcribed and analysed by an investigator using framework analysis, counts, and content analysis.

### Important amendments to protocol

The trial protocol was published on 21 September 2022. NHS organisational pressures on emergency and trauma services preventing timely hospital admission from the emergency department after injury and therefore access by researchers to participants, led the investigators to amend the published eligibility criteria to remove the inclusion criteria that participants must be able to receive study intervention within 12 h of hospital admission (NHS research ethics approval: 9 November 2022). In response to unanticipated site shortages of levobupivacaine, we removed reference to this specific local anaesthetic drug from our protocol to allow use of any long-acting local anaesthetic for ESP block (NHS research ethics approval: 22 November 2022).

## Results

### Participant flow

**Fig 1:**
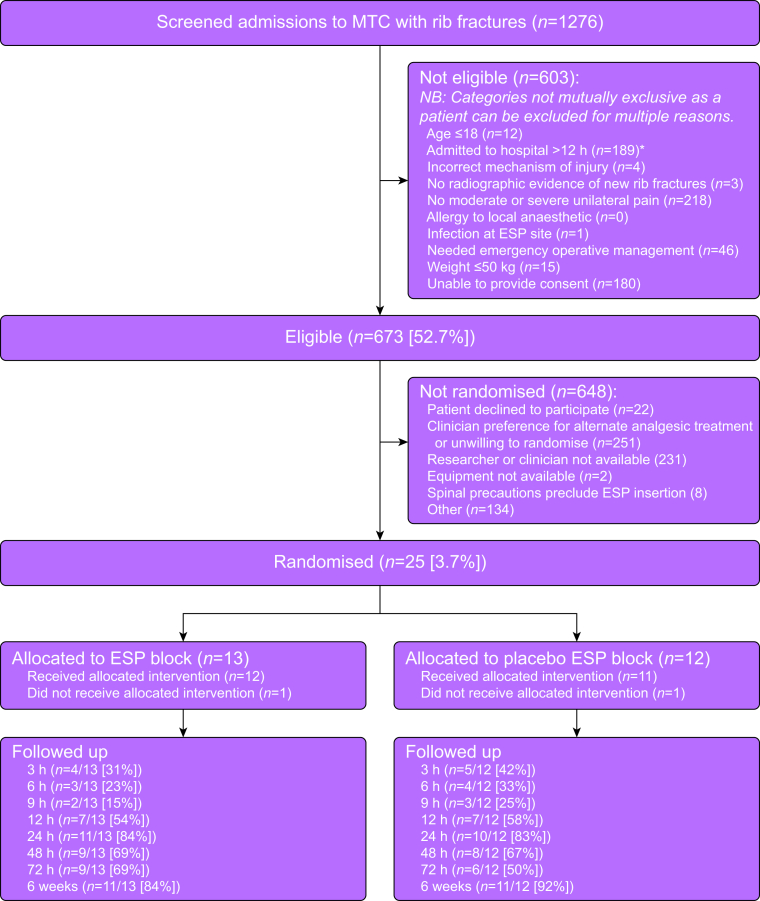
CONSORT participant flow diagram. ESP, erector spinae plane; MTC, major trauma centre. ∗Removed as an inclusion criteria after study commencement.

A CONSORT flow diagram of all potential and recruited participants is shown in Figure 1, including reasons for ineligibility. A total of 1276 patients were screened of whom 673 were eligible for recruitment. Twenty-five participants were enrolled to the trial and randomised between 08 August 2022 and 10 January 2024. The final participant follow-up was completed on 21 February 2024. The trial was terminated because of completion of planned funding. The median (IQR) time from injury to trial randomisation was 13 (29 [0–94]) h. The median time from trial randomisation to receipt of trial intervention was 3 (5 [0–45]) h.

### Primary and secondary feasibility outcomes

Trial recruitment at each site is summarised in Table 2. The overall recruitment rate was 0.69 participants randomised per site per month. The eligibility rate was 52.7% (673/1276). Of eligible potential participants 37.3% (251/673) were not subsequently enrolled because of treating clinician preference for another analgesic technique (such as TEA) to manage their post-injury acute pain. A further 35.6% (231/673) of eligible potential participants were not enrolled because of the unavailability of either researchers or clinicians to undertake research activities or site trial interventions in a clinically timely manner. The trial consent rate was 3.7% (25/673). The retention rate to 6-week follow-up was 20/25 (80%).

**Table 2:** Trial recruitment by site. ∗Pre-recruitment site screening identified the relative extent to which erector spinae plane (ESP) block formed part of the existing clinical care pathway for the analgesic management of adults with rib fractures alongside alternative analgesic and regional anaesthetic techniques such as serratus plane block, paravertebral block, or thoracic epidural analgesia. ^†^Open to recruitment 1 February 2023 to 31 January 2024. ^‡^Open to recruitment 14 July 2022 to 31 January 2024 with recruitment on hold 18 November 2022 to 4 January 2023 (pending protocol amendments as detailed in Methods) and 14 July 2023 to 27 October 2023 (pending locally-approved extension to recruitment window). ^¶^Open to recruitment 1 February 2023 to 31 January 2024.

Site name	Site characteristic∗	Duration of recruitment (months)	Randomised participants (*n*)	Recruitment rate (participants randomised/month)
The Royal London Hospital, Bart’s Health NHS Trust	Low ESP use	12^†^	2	0.17
Queen’s Medical Centre, Nottingham University Hospitals NHS Trust	Medium ESP use	13^‡^	9	0.69
Manchester Royal Infirmary, Manchester University NHS Foundation Trust	High ESP use	12^¶^	14	1.17

### Secondary candidate clinical outcome measures

Participants’ baseline characteristics are summarised in Table 3. Hyperacute and acute candidate clinical outcome measure results, including extent of missing data, are shown in Table 4, Table 5, respectively. Complete data on VAS pain scores at 24 h after trial intervention was available for 21/25 (84%) of participants. Six-week follow-up data are presented in Table 6.

**Table 3:** Participant characteristics at randomisation by group allocation. ESP, erector spinae plane; IQR, inter-quartile range; sd, standard deviation; VAS, visual analogue scale. ∗Respiratory co-morbidities defined as presence of asthma, chronic obstructive pulmonary disease, bronchiectasis, interstitial lung disease, lung cancer or obstructive sleep apnoea.

	ESP group (*n*=13)	Placebo ESP group (*n*=12)
Age (yr), mean (range)	62.7 (18–85)	57.8 (36–71)
Female, *n* (%)	5 (38)	5 (42)
Injury pattern, *n* (%) (unless otherwise stated)		
Presence of bilateral fractures	0	0
Number of rib fractures (mean [sD])	5 (3)	5 (2)
Radiological flail segment	5 (38)	1 (8)
Concurrent haemothorax, pneumothorax, or both	8 (62)	7 (58)
Concurrent non-thoracic traumatic injuries	8 (62)	7 (58)
Pre-existing respiratory co-morbidity∗	3 (23)	4 (33)
Pain (mm on 100 mm visual analogue scale), mean (sd)		
Chest wall pain intensity at rest	43 (25)	62 (18)
Chest wall pain intensity on maximal inspiratory effort	71 (16)	77 (23)
Chest wall pain intensity on maximal cough effort	79 (16)	88 (11)
Respiratory function, mean (sD)		
Forced vital capacity (L)	1.64 (0.8)	1.83 (0.36)
Forced expiratory volume in 1 s (L)	1.31 (0.7)	1.58 (0.35)
Peak expiratory flow rate (L min^−1^)	127 (124)	185 (151)
Post-injury analgesia received before randomisation, *n* (%) (unless otherwise stated)	*n*=10	*n*=11
Paracetamol	9 (90)	10 (91)
Non-steroidal anti-inflammatory agent	0	2 (18)
Ketamine	0	1 (9)
Oral morphine equivalents (mg), median (IQR)	10 (7–20)	26 (15–60)

**Table 4:** Hyperacute (i.e. 3, 6, 9, and 12 h after receipt of trial intervention) candidate clinical outcomes by group allocation. Extent of missing data presented as *n* (%). ESP, erector spinae plane; FEV1, forced expiratory volume in 1 s; FVC, forced vital capacity; PEFR, peak expiratory flow rate; sD, standard deviation; VAS, visual analogue scale.

	ESP group (*n*=13)	Placebo ESP group (*n*=12)
Supplemental oxygen requirement, *n* (%)		
3 h	2 (15)	4 (33)
Missing	9 (69)	7 (58)
6 h	2 (15)	3 (25)
Missing	10 (77)	8 (67)
9 h	2 (15)	3 (35)
Missing	11 (85)	9 (75)
12 h	4 (31)	4 (33)
Missing	6 (46)	5 (42)
FVC (L)/FEV1 (L)/PEFR (L min^−1^), mean (sd)		
3 h	2.59 (0.35)/1.66 (0.16)/269 (121)	1.26 (0.46)/0.87 (0.21)/145 (3)
Missing	11 (85)	10 (83)
6 h	3.28/2.71/652	1.25 (0.06)/0.98 (0.36)/130 (47)
Missing	12 (92)	10 (83)
9 h	—	—
Missing	13 (100)	12 (100)
12 h	1.33 (1.14)/0.92 (0.77)/199 (146)	1.45 (0.66)/1.2 (0.66)/178 (207)
Missing	9 (69)	3 (25)
Chest wall pain intensity at rest/maximal inspiration/maximal cough (100 mm VAS), mean (sd)		
3 h	37 (31)/44 (11)/61 (32)	33 (22)/78 (19)/77 (32)
Missing	10 (77)	9 (75)
6 h	20/20/5	43 (17)/57 (2)/59 (5)
Missing	12 (92)	10 (83)
9 h	—	—
Missing	13 (100)	12 (100)
12 h	16 (9)/47 (22)/83 (18)	35 (12)/55 (15)/72 (27)
Missing	8 (62)	8 (67)

**Table 5:** Acute (i.e. 24, 48, and 72 h after receipt of trial intervention) candidate clinical outcomes by group allocation. Extent of missing data presented as *n* (%).ESP, erector spinae plane; FEV1, forced expiratory volume in one second; FVC, forced vital capacity; IQR, inter-quartile range; PEFR, peak expiratory flow rate; sD, standard deviation; VAS, Visual Analogue Scale. ∗Complications of regional anaesthetic procedure include local anaesthetic systemic toxicity, bleeding at catheter insertion site, infection at catheter site or catheter dislodgement. ^†^Additional regional anaesthetic procedures include peripheral, fascial plane or neuraxial analgesic technique performed for relief of chest wall pain after trial intervention at the discretion of the treating clinician.

	ESP group (*n*=13)	Placebo ESP group (*n*=12)
FVC (L)/FEV1 (L)/PEFR (L min^−1^), mean (sD)		
24 h	1.66 (0.66)/1.37 (0.68)/196 (120)	1.52 (0.54)/1.32 (0.57)/168 (160)
Missing	3 (23)	6 (50)
48 h	2.26 (0.80)/2.07 (0.92)/307 (205)	2.06 (0.29)/1.65 (0.54)/244 (210)
Missing	7 (54)	8 (67)
72 h	1.90 (1.11)/1.57 (0.89)/238 (176)	1.44 (0.63)/0.91 (0.56)/150 (81)
Missing	6 (46)	8 (67)
Chest wall pain intensity at rest/maximal inspiration/maximal cough (100mm VAS), mean (sd)		
24 h	26 (21)/44 (29)/61 (33)	43 (30)/65 (24)/75 (32)
Missing	2 (15)	2 (17)
48 h	24 (16)/52 (17)/74 (10)	43 (37)/48 (40)/46 (38)
Missing	7 (54)	5 (42)
72 h	31 (35)/60 (26)/73 (25)	35 (30)/53 (13)/74 (27)
Missing	4 (31)	7 (58)
Opioid consumption in oral morphine equivalents (mg), median (IQR)		
24 h	30 (15–40)	15 (0–110)
Missing	2 (15)	2 (17)
48 h	20 (10–50)	20 (7.5–57.5)
Missing	5 (38)	4 (33)
72 h	20 (7.5–32.5)	20 (2.5–95)
Missing	4 (31)	7 (58)
Opioid side-effects: nausea or vomiting/pruritus/opioid-induced sedation, *n* (%)		
24 h	4 (31)/2 (15)/0	2 (17)/6 (50)/2 (17)
Missing	2 (15)	3 (25)
48 h	1 (8)/1 (8)/0	0/4 (33)/1 (8)
Missing	5 (38)	4 (33)
72 h	2 (15)/2 (15)/0	0/3 (25)/1 (8)
Missing	5 (38)	7 (58)
Complication of regional anaesthesia∗, *n* (%)		
24 h	1 (8)	0
Missing	2 (15)	2 (17)
48 h	1 (8)	0
Missing	5 (38)	4 (33)
72 h	0	0
Missing	4 (31)	7 (58)
Additional regional anaesthetic procedures performed^†^, *n* (%)		
24 h	1 (8)	3 (25)
Missing	2 (15)	2 (17)
48 h	0	2 (17)
Missing	5 (38)	5 (42)
72 h	1 (8)	0
Missing	4 (31)	7 (58)
Diagnosis of pneumonia, *n* (%)		
24 h	1 (8)	0
Missing	2 (15)	2 (17)
48 h	0	1 (8)
Missing	5 (38)	4 (33)
72 h	0	0
Missing	4 (31)	7 (58)
Admission to critical care, *n* (%)		
24 h	0	1 (8)
Missing	2 (15)	2 (17)
48 h	0	1 (8)
Missing	5 (38)	4 (33)
72 h	0	1 (8)
Missing	4 (31)	7 (58)

**Table 6:** Six-week follow-up candidate clinical outcomes by group allocation. ESP, erector spinae plane; sd, standard deviation.

	ESP group (*n* = 13)	Placebo ESP group (*n* = 12)
Mortality		
*n* (%)	0	0
Missing, *n* (%)	2 (15)	1 (8)
Length of hospital stay		
Days, mean (sd)	11.7 (8.6)	6.9 (4)
Missing, *n* (%)	2 (15)	1 (8)
Discharge destination		
Home, *n* (%)	10 (77)	11 (92)
Healthcare facility/institution, *n* (%)	1 (8)	0
Missing, *n* (%)	2 (15)	1 (8)

### Embedded qualitative assessment

Evaluation of interviews from patients who declined participation (*n*=2), trial participants (*n*=7), researcher delivery staff (*n*=2), trial management group members (*n*=3), and independent clinical staff (*n*=2) identified three main themes consistently reported as impeding trial recruitment and data completeness. In addition, qualitative feedback was gathered on the importance of the research, acceptability of the trial, effectiveness of their blinding to group allocation by means of sham ESP block and infusion device.

#### Theme 1: participant perceptions of the trial, the intervention, and allocation integrity

The inclusion of a placebo ESP intervention as the trial comparator arm was reported as weakening clinical equipoise because of concern of treating clinicians that participants randomised to this group would be unfairly under-treated with analgesia, and a barrier to consent from potential participants. Trial procedures and materials attempted to mitigate this by reassuring participants and clinicians that patients in both arms should receive all other analgesia as normal as dictated by local chest wall injury policy, and that participants randomised to either treatment group were able to receive additional regional anaesthetic procedures after randomisation if deemed necessary by the treating clinician. Despite this protocolisation, and although inclusion of a placebo intervention is methodologically supported in trials of analgesic interventions, interviews suggest this incurred a significant deleterious impact on the willingness of both patients with rib fractures and acute post-injury pain, and their treating clinicians, to enrol in the trial and undergo randomisation. One clinician (C05) said, ‘*I just do not think these [ESP blocks] work as well, and at this hospital where we’re already seeing the worst cases, it doesn’t make sense to me to not use an epidural*’. This qualitative feedback accords with our finding that 273/673 (40.6%) of eligible potential participants were not subsequently enrolled because of either participant or clinician refusal.

The attempt to blind participants and outcome assessors to group allocation (by use of sham ESP catheters and infusion devices in the placebo control arm) was reported as ineffective by several interviewees. One participant (P017) said, ‘*It was immediately obvious that I was in the ‘fake’ arm. The machine was clearly turned off and the nurses paid zero attention to it*’. Another (P018) recalled, ‘*Initially I didn’t realise it wasn’t a real injection, but then the tape and plastic fell off and the doctor explained it had been a fake*’. Inadvertent unblinding occurred by two mechanisms: either by clinical staff (who were not blinded) accidentally revealing group allocation during routine care or by participants and outcome assessors themselves identifying that the sham infusion device was in an ‘off’ mode and therefore not administering local anaesthetic infusion. Maintaining fidelity of blinding for 72 h on busy inpatient wards with multiple clinical staff caring for participants was reported by researchers as unfeasible.

Most participants reported no fixed views about rib fracture management and accepted clinical recommendations. (P011: ‘*they know what they are doing and they suggested this and I wanted to give it a go*’). Those who declined typically cited a wish to avoid hospital or procedures (PD02: ‘*I really don’t like needles, and I didn’t plan to stay in hospital*’). Despite mixed experiences, participants remained positive and would recommend the trial, even if they had received placebo.

#### Theme 2: access of researchers and trained clinicians to potential participants

At recruiting sites without pre-existing embedded use of ESP block for rib fracture analgesia in local clinical practice (for example because local practice favoured TEA), the availability of anaesthetists with necessary expertise to perform ESP block and catheter insertion, and familiarity with trial procedures, in a timely manner for trial purposes was reported by staff as a significant barrier to recruitment. For example, one clinician (C06) commented, ‘*You needed the right anaesthetist, at the right time - and those people often weren’t available within the timescale we had*’. Clinicians described greater familiarity with single-shot techniques and noted that the procedural skill required was not routinely held across the workforce. This qualitative feedback is supported by our finding that among eligible potential participants 231/673 (34.3%) were not enrolled because of unavailability of either a member of the research team to execute trial processes or a trial-trained clinician to site the trial intervention.

#### Theme 3: data collection in a hyperacute setting

Researchers reported that completion of follow-up assessments in the hyperacute phase (3, 6, 9, and 12 h after trial intervention) was difficult. With acute follow-ups occurring at unsociable times, one staff member (C04) noted, ‘*You’d have needed someone available at all hours —3am, 6am—it wasn’t feasible*’. Overnight cover was impractical because of low patient numbers and high costs for trained staff. This was as a result of these time points falling outside normal office hours and because of their complexity (e.g. bedside spirometry), which necessitated performance by trained researchers rather than clinical staff. This feedback is in keeping with unacceptably high degrees of missing data for 100 mm VAS pain scores and spirometry and reported in Table 4.

Some participants reported they found the questionnaires repetitive and irrelevant. One (P010) noted, ‘*They asked me if I felt like I could run for the bus, felt like I’d been hit by the bus more like*’. Research staff acknowledged each time point required around 30 min, and more concise, targeted tools were recommended. Self-reported methods of outcome assessment (e.g. text messages) were explored. Research staff questioned the feasibility of such methods given the acuity and injury burden of the traumatic rib fracture population. Patient views were mixed; some (*n*=4) felt self-reporting was unfeasible, while others (*n*=2) thought it may be possible with appropriate support.

## Discussion

The main finding of this feasibility study comparing ESP with placebo ESP for early analgesia in adults with rib fractures after blunt chest wall injury and undertaken to inform the viability and design of potential future definitive clinical trials on this topic, was that current study design is not feasible to up-scale to a definitive trial, and a large trial will only be possible with substantial protocol revision as suggested by our qualitative assessments.

The main definitive trial protocol recommendations arising from this work are summarised in Table 7. Although our reported recruitment and retention rates (primary feasibility outcomes) appear modest, particularly in comparison to single-centre assessments of perioperative interventions which can recruit at higher rates, Jacques and colleagues^15^ report the median (IQR [range]) recruitment of National Institute for Health Research (NIHR) Health Technology Assessment trials from 1997 to 2020 to be 0.85 (0.39–2.49 [0.01–57.75]) participants/site/month with a retention rate of 89%. Furthermore, the same authors report inclusion of a placebo control (as opposed to an active control arm) as negatively impacting speed of recruitment. Comparable multicentre trials of acute interventions in patients with rib fractures have recruited at similar rates.^16^^,^^17^

**Table 7:** Proposed substantial revisions to any subsequent definitive trial PICO examining the clinical effectiveness of ESP blocks in patients with rib fractures, as informed by ESPEAR. ESP, erector spinae plane; ESPEAR, Erector Spinae Plane block with fascial plane catheter for the Early Analgesia of Rib fractures in trauma trial; PICO, population, intervention, comparator, and outcomes.

	ESPEAR pilot protocol	Barriers identified	Modified methodology for definitive trial	Justification for modification
Population	Adults with one or more rib fractures able to receive trial intervention within 12 h of hospital admission.	Researchers and trained clinicians unable to site intervention within stated time period, preventing recruitment.	Adults with one or more rib fractures, with no specification of timeframe for trial intervention	Organisational pressures on emergency and trauma services prevented researchers gaining timely access to participants.
Intervention	ESP block with insertion of fascial plane catheter and infusion of local anaesthetic for 72 h alongside multimodal analgesia.	Lack of trained anaesthetists able to perform technique in timely manner on potential participants.	Single-shot ESP block alongside multimodal analgesia.	In greater clinical use, wider staff training in single-short analgesia including non-anaesthetic staff (e.g. emergency care physicians)
Comparator	Placebo ESP block using sham fascial plane catheter and sham infusion of local anaesthetic for 72 h alongside multimodal analgesia.	Lack of clinical equipoise, patient reticence to consent to inclusion. Poor fidelity of participant and outcome assessor blinding.	Multimodal analgesia only.	Established standard of care in patient pathway. No requirement for training in sham ESP procedure.
Outcomes	Assessed at baseline then 3, 6, 9, 12, 24, 48, 72 h, and 6 weeks after trial intervention.	Significant out-of-hours burden of data collection resulting in large degree of data missingness in hyperacute time points.	Baseline before intervention, 24 h, 30 days.	Reduced burden on participants and researchers. Improved data completeness.

Recruitment to a larger trial will be optimised by meticulous screening of NHS sites to identify those already delivering ESP block in their local clinical care packages for rib fracture analgesia. Although ESP block is a core competency within the UK regional anaesthetic curriculum and endorsed as a ‘Plan A’ block by RA-UK, this study has shown that it is not yet sufficiently embedded in practice at MTCs to support timely trial delivery. In sites where local practice favoured thoracic epidural or opioid-based regimes, the availability of anaesthetists with the necessary skills and confidence to insert ESP catheters was limited. Clinicians described greater familiarity with single-shot ESP techniques and viewed catheter-based approaches as requiring a higher level of technical experience. This skill gap, combined with the urgent nature of rib fracture management—often requiring intervention within hours of injury and outside of standard working hours—meant that both researcher and clinician availability were frequently insufficient to support enrolment. A future definitive trial should therefore prioritise site selection where ESP catheter techniques are already standard care, and where appropriate anaesthetic and research support is reliably available.

The absence of national guidelines on analgesic management of patients after blunt chest wall injury, and the substantial heterogeneity of local clinical practice guidelines in NHS major trauma centers,^5^ means active site screening will be vital to implement a definitive trial on this topic. The formation of a UK-wide representative ‘link person’ network by RA-UK, the professional specialist society for regional anaesthesia, may assist in candidate site identification and enable delivery of a definitive trial in a timely and pragmatic fashion. Given the high rate of non-enrolment (37.3% of potential participants) because of treating clinician preference for alternative regional anaesthetic techniques (e.g. TEA), site feasibility in a definitive trial must carefully assess underlying clinician treatment preferences alongside skill and training in siting of ESP blocks.

Our study data displayed very high rates of data missingness in the hyperacute outcome assessment time points of 3, 6, 9, and 12 h after trial intervention. This arose as a result of the trial interventions being sited by clinicians during daytime care and therefore these research time points largely fall outside of standard working hours for research teams. Although ESP block may be expected to exert a beneficial effect within 20 min of institution and for as long as the fascial plane catheter remains *in situ*, capturing this early analgesic benefit using researcher-administered outcome measures is not feasible. Possible alternatives in a definitive trial are to use patient diaries to capture out-of-hours data, or simple outcome assessments that can be accurately administered by nursing or medical staff without prior training. Wireless monitoring of respiratory function by remote spirometry, recently reported in the rib fracture population,^18^ may also usefully increase respiratory data completeness. Recently published core outcome sets of pain-related instruments for assessing effectiveness of analgesic interventions should be incorporated into a definitive trial protocol.^19^

Strengths of this multicentre feasibility study include the pre-published study protocol, predefined trial progression criteria, central randomisation and data management and pragmatic accommodation of site-specific multimodal analgesia to all participants. Future research must consider the extent to which factors likely to affect block effectiveness (for example, the use of high-volume intermittent bolus regimes) should be standardised as part of trial processes. Although such protocolisation would strengthen the test of efficacy for the intervention, this would come at a cost of loss of pragmatism and external validity of trial results for the wider clinical community. A large-scale trial applying a pragmatic approach to the intervention (accepting a variety of infusion regimes for ESP blocks) could apply sub-group analyses to distinguish treatment effects between different regimes.

The trial tested the practicality of a placebo comparator arm, together with qualitative assessment of participant and outcome assessor blinding, all of which have strong methodological justification when evaluating the effectiveness of analgesic interventions by patient-reported outcome measures. The trial clearly demonstrated that blinding effectiveness was reported as poor by participants and that a definitive trial must carefully consider whether the methodological benefits of blinding outweigh the costs (in logistics and finances) of developing a more sophisticated system of blinding with greater fidelity than the system trialled in this present work.

In conclusion, this multicentre feasibility randomised trial piloted a protocol for the assessment of the clinical effectiveness of ESP block and catheter in adults with rib fractures. The study reports an ‘amber’ feasibility result, defined as not feasible for definitive study without protocol modification. This was determined by recruitment rate, retention rate and qualitative feedback from participants and staff. We offer recommendations on protocol modification to maximise recruitment, retention and data completeness, should a definitive trial on this topic be undertaken.

## Authors’ contributions

Substantial contributions to conception and design, acquisition of data, or analysis and interpretation of data; drafting of the article or revising it critically for important intellectual content; final approval of the version to be published; agree to be accountable for all aspects of the work: all authors.

## Funding

The National Institute for Health Research (NIHR) (Research for Patient Benefit programme Call 42, [project reference: NIHR202195]). The views expressed are those of the authors and not necessarily those of the NIHR or the UK Department of Health and Social Care.

## Declarations of interest

DWH is an Associate Editor of the *British Journal of Anaesthesia* and an RA-UK Board member. MLC is a National Institute for Health Research (NIHR) Senior Investigator. The remaining authors declare that they have no conflicts of interest.
